# Multi-Modal, Remote Breathing Monitor

**DOI:** 10.3390/s20041229

**Published:** 2020-02-24

**Authors:** Nir Regev, Dov Wulich

**Affiliations:** School of Electrical and Computer Engineering, Ben-Gurion University of the Negev, Beer-Sheva 8410501, Israel; dov@ee.bgu.ac.il

**Keywords:** vital signs, IR-UWB radar, micro-Doppler, optical flow, spectral estimation

## Abstract

Monitoring breathing is important for a plethora of applications including, but not limited to, baby monitoring, sleep monitoring, and elderly care. This paper presents a way to fuse both vision-based and RF-based modalities for the task of estimating the breathing rate of a human. The modalities used are the F200 Intel^®^ RealSense^TM^ RGB and depth (RGBD) sensor, and an ultra-wideband (UWB) radar. RGB image-based features and their corresponding image coordinates are detected on the human body and are tracked using the famous optical flow algorithm of Lucas and Kanade. The depth at these coordinates is also tracked. The synced-radar received signal is processed to extract the breathing pattern. All of these signals are then passed to a harmonic signal detector which is based on a generalized likelihood ratio test. Finally, a spectral estimation algorithm based on the reformed Pisarenko algorithm tracks the breathing fundamental frequencies in real-time, which are then fused into a one optimal breathing rate in a maximum likelihood fashion. We tested this multimodal set-up on 14 human subjects and we report a maximum error of 0.5 BPM compared to the true breathing rate.

## 1. Introduction

Vital signs extraction has been a research topic both in the computer vision and radar research community. The computer vision-based algorithms are tackling this problem from two different angles: One is the color-based algorithms [1,2,3,4], which capture the minute color variation of the human skin during a heartbeat [5]. Color based algorithms are primarily used for heartbeat estimation which is not the scope of this paper. The other is known as motion magnification [6] that magnify minute movements in a video. This is used mainly for heartbeat but can be used also for breathing rate estimation.

Intel’s RealSense camera was used in [7] to estimate the heartrate of a human subject. They used the infrared (IR) channel for estimating the heartrate. The depth channel was used as well for estimating the pose of the human head. The use of optical flow for breathing estimation was presented in [8]; however, the detailed algorithm and result performance was not reported. Finally, in [9] we describe in details the algorithm to reliably extract breathing from a RGB video alone in real-time.

Lately, biological signals monitoring utilizing uDoppler has been the topic of the research community. Respiration rate extraction with a pulse-Doppler architecture is presented in [10]. The wavelet transform was used in [11] to overcome the Discrete Fourier Transform (DFT) resolution insufficiency, and in [12], the chirp Z transform was used on a IR-UWB radar echos to extract respiration rate. The same transform was used in [13] coupled with an analytical model for the remote extraction of both respiration and heartrate. Moreover, they verified the validity of a model in which the thorax and the heart are considered vibrating-scatterers, such that the total uDoppler return is a superposition of two sinusoids with different frequencies and amplitudes in which, the breathing frequency is smaller than the heartrate, and its amplitude is much larger.

In this paper, we add to the reported results in [9] and show that breathing information also lies in the depth and radar signals. Coupled with the available RGB information, we can improve the reported accuracy results by more than twofold. We will use optical flow tracking coupled with a sinusoidal detector to determine if the optical flow track is sinus-like, exactly as presented in [9]. Each tracked point of interest coordinate change through time is considered a separate signal. In addition, at these specific coordinates of interest, the depth values are extracted from the depth channel. All these signals including the analyzed radar return are then fed into a maximum likelihood estimator to produce one optimal breathing rate. In Section 2, we describe the radar set-up, and in Section 3, we describe the algorithm outline. The method is outlined in Section 4, followed by the experiments outline and the results in Section 5. We conclude the paper in Section 6.

## 2. Radar Measurement Setup

**Figure 1:**
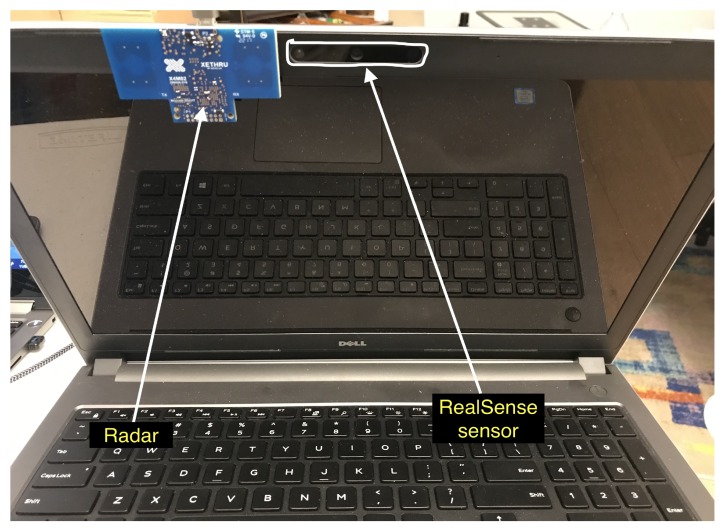
Xethru X4 radar mounted to a laptop with RealSense sensor on it.

**Figure 2:**
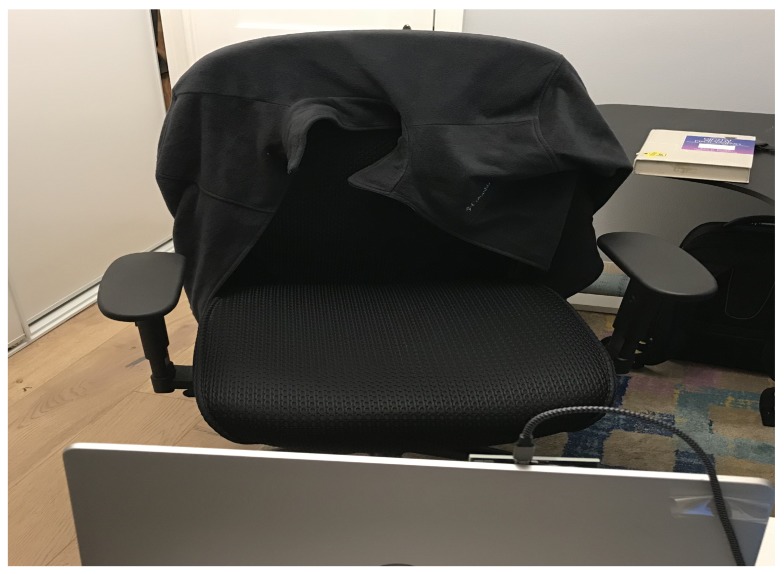
Subject under test will be sitting on this chair infront of the radar and RealSense sensor.

The radar measurement setup is given in Figure 1 and Figure 2. We use a IR-UWB impulse XeThru X4 radar module that transmits on a human subject (hereinafter “subject”). The raw data is collected at the PC through a USB interface and fed into the algorithm which is running real time. The radar is synced to the F200 Intel^®^ RealSense^TM^ RGBD sensor. The radar operating parameters is given in Table 1. This single setup cost is ~$400. The cost can be reduced substantially after system design and large quantities discounts.

## 3. Algorithm Outline

For the sake of brevity, we repeat the main ideas of the algorithm outline. The reader is referred to [9] for a more detailed description. The first component of the system is the F200 Intel^®^ RealSense^TM^ sensor, which is capturing image frames at a resolution of 640×480 pixel, and at a rate of 10 frames per second (FPS). Frame by frame, we extract *N* feature points using Shi and Tomasi [14] algorithm. Next, optical flow tracking [15] is used, and we save the last three x, y, depth coordinates for each feature point. The depth value at these specific x,y coordinates is read from the sensor’s depth channel. We use each feature point at the last three x, y, depth values to estimate a fundamental frequency for each of the points, producing a 3N×1 vector of estimated fundamental frequencies. The estimation is computed iteratively based only on the last three samples using the reformed Pisarenko harmonic decomposition (RPHD) algorithm [16]. Next, a sine detector using a generalized likelihood ratio test is executed on each signal to detect which of the signals follows a sinus-like pattern. The signals that pass this test are kept while separate breathing rate is estimated from the synced radar signal in a way we will describe further in the paper. Last, all the plurality of breathing rates are fused in a ML fashion producing one optimal breathing rate estimation result.

## 4. Method

### 4.1. Model

The assumed model due to breathing is a sinus-like motion on the axes, namely, x-axis, y-axis, and the depth axis. Therefore, denoting by 3N the number of signals acquired from the RGBD sensor. We can write

(1) $$z_{i}\left(k\right)=a_{i}\sin\left(\omega_{b}k+\varphi_{b}\right)+v_{i}\left(k\right),\;i=0,\cdots ,3N-1,$$

where $\omega_{b}$ is the breathing angular frequency in rad/sec, $v_{i}\left(k\right)∼N\left(0,\sigma^{2}\right)$ and $z_{i}\left(k\right)$ is the *k*-th sample of a sinusoid on a certain axis, $k=0,\cdots K_{i}-1$ and $K_{i}$ is the number of samples observed at the *i*-th feature of interest. The noise is assumed to be i.i.d in space and time.

### 4.2. Acquiring Time Domain Signals from Rgbd

The body moves due to the breathing. A camera is projecting this 3D movement into the image plane. Our goal is to acquire and track enough moving points on the image plane and observe the change in coordinates and depth through time, and, in real-time, estimate the motion’s fundamental frequencies. Thus, we get plurality of estimations for the breathing rate. These are all fused in an optimal manner to get one estimation of $\omega_{b}$.

The signals acquisition and tracking is done using the algorithms of Shi-Tomasi and Lucas-Kanade [14,15], respectively.

**Figure 3:**
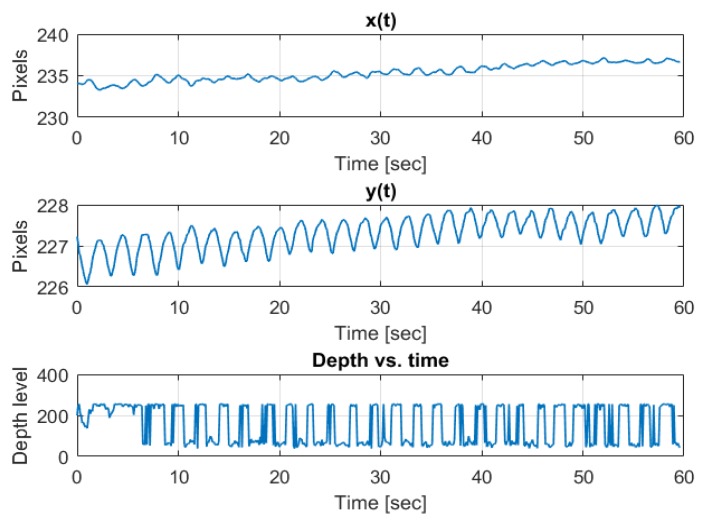
Pre band-pass filtering example of three axes (x, y, depth) breathing pattern.

**Figure 4:**
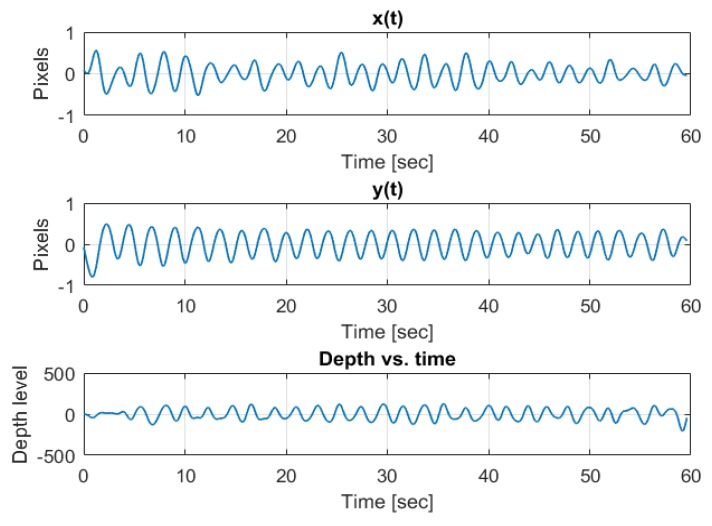
Post band-pass filtering example of three axes (x, y, depth) breathing pattern.

An example of a coordinate change through time of an arbitrary tracked feature point while the author was breathing in front of the camera is depicted in Figure 3. We observe the sinus-like coordinate change carried on a trend line due to small shifts in the body location as well as small biases in the optical flow tracking algorithm. The depth is carried on a DC term, and is very square-wave-like due to low resolution of depth quantization. The same signals passed through a band-pass filter with cutoff frequencies at the respiration band is depicted in Figure 4.

Therefore, as depicted in Figure 3, the observed signal can be written as

(2) $${\tilde{z}}_{i}\left(k\right)=c_{i}k+d_{i}+a_{i}\sin\left(\omega_{b}k+\varphi_{b}\right)+v_{i}\left(k\right),$$

where $c_{i},d_{i}\in R$ are the trend parameters for the *i*-th signal.

Thus, we will estimate those two trend parameters for each of the 3N signals, and then subtract the trend from the observed signal, thus getting the desired model sa in (1).

### 4.3. Estimating the Trend

We use the recursive least squares (RLS) algorithm [17] (pp. 566–571) to estimate the trend real time, taking only the last three samples, namely, *k*, k−1, k−2 into account. This algorithm is executed on all signals. For in-depth breakdown of the algorithm the reader is referred to [9].

Next, we de-trend the signals by subtracting the trend from ${\tilde{z}}_{i}\left(k\right)$, to get

(3) $$z_{i}^{-}\left(k\right)=a_{i}\sin\left(\omega_{b}k+\varphi_{b}\right)+v_{i}^{-}\left(k\right),\;i=0,\cdots ,3N-1,$$

where $v_{i}^{-}\left(k\right)$ is white noise with variance $\sigma_{i}^{2}$ representing the estimation error.

### 4.4. Estimating the Fundamental Frequency, $\omega_{b}$

We chose to use the RPHD algorithm [16] for iteratively estimating the fundamental frequency $\omega_{b}$.

The algorithm, fed by only the last three samples, iteratively and asymptotically efficiently estimates the fundamental frequency of a sinusoid as shown in [16].

The algorithm starts by ”waiting” for the first three frames to come in, and then it is refining the estimation iteratively with each new available sample.

This estimator’s variance can be shown to be [9,16]

(4) $$\begin{array}{cc} VAR_{i}\left[{\hat{\omega }}_{b}\right] & =\frac{\cos^{2}\left(2\omega_{b}\right)+\cos^{2}\omega_{b}}{SNR^{2}\left(K_{i}-2\right){\left[2+\cos\left(2\omega_{b}\right)\right]}^{2}\sin^{2}\omega_{b}}\\ & +\frac{1}{SNR{\left(K_{i}-2\right)}^{2}\sin^{2}\omega_{b}}\\ & +\frac{3+4\cos\left(2\omega_{b}\right)-cos\left(4\omega_{b}\right)}{4SNR^{2}{\left(K_{i}-2\right)}^{2}{\left[2+\cos\left(2\omega_{b}\right)\right]}^{2}\sin^{2}\omega_{b}}. \end{array}$$

**Figure 5:**
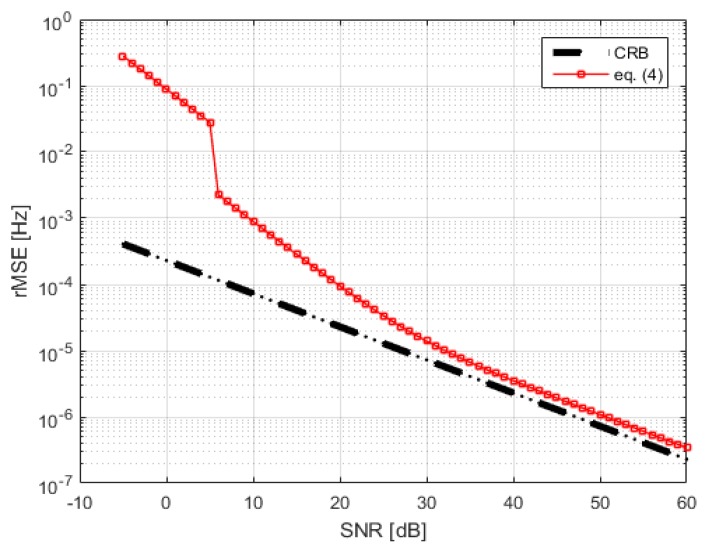
CRB and Equation (4) vs. SNR for K = 1000.

As seen in (4), the estimator’s variance is a function of the number of samples and the SNR. Therefore, we need to estimate $\sigma_{i}^{2}$. This is done by computing the spectral power outside the respiration frequency band. We compare the estimator’s variance to the CRB in Figure 5. This variance calculation is used in the ML fusion step.

### 4.5. Generalized Likelihood Ratio Test (Glrt)

All estimated fundamental frequencies that are outside the respiration band are immediately discarded. The signals that survive this are tested to be oscillating, or in other words, to follow the model given in (3). Thus, we look at the following two hypotheses,

(5) $$\begin{array}{ccc} H_{0} & : & z_{i}^{-}\left(k\right)=v_{i}^{-}\left(k\right)\\ H_{1} & : & z_{i}^{-}\left(k\right)=a_{i}\sin\left(\omega_{b}k+\varphi_{b}\right)+v_{i}^{-}\left(k\right), \end{array}$$

i=0,⋯,3N−1, which is solved by applying a quadrature matched filter on each signal [18] (pp. 262–268)

(6) I0iω^biH1≷H0γi,

where $I_{0_{i}}\left({\hat{\omega }}_{b_{i}}\right)$ is the well-known periodogram and $\gamma_{i}$ and is the test threshold. $I_{0_{i}}\left({\hat{\omega }}_{b_{i}}\right)$ is given by

(7) $$I_{0_{i}}\left({\hat{\omega }}_{b_{i}}\right)=\frac{1}{\sqrt{K_{i}}}\sum_{k=0}^{K_{i}-1}\left[{\left(z_{i}^{-}\left(k\right)\sin\left({\hat{\omega }}_{b}k\right)\right)}^{2}+{\left(z_{i}^{-}\left(k\right)cos\left({\hat{\omega }}_{b}k\right)\right)}^{2}\right].$$

The per signal threshold, $\gamma_{i}$ is derived by fixing a constant probability of false alarm, and using Neyman-Pearson’s theorem, and is given by [18] (Equation (7.26))

(8) $$\gamma_{i}=-\sigma_{i}^{2}\ln P_{FA}.$$

The periodogram and its threshold are updated iteratively with each sample that comes in. The signals that do not meet the threshold are discarded, the rest of the signals’ estimated fundamental frequencies are fused, as presented in Section 4.7.

### 4.6. Radar Based Breathing Extraction

We denote by X(k) the slow vs. fast-time matrix of size $N_{T}\times N_{rg}$, where $N_{T}$ is the number of frames, each frame is a row in this matrix where *k* corresponds to the current frame or the last row. Each frame sample represent a different fast-time bin, and is called a range-gate. The number of range gates is denoted by $N_{rg}$. Therefore, there are $N_{rg}$ slow-time signals corresponding to the columns of this matrix.

All of these slow-time signals are band-pass filtered from 0.1 to 5 Hz. Next, a pre-FFT step of Hanning windowing is applied and then each matrix column is transformed to the Fourier domain by FFT. The matrix we get is a range-Doppler map.

**Figure 6:**
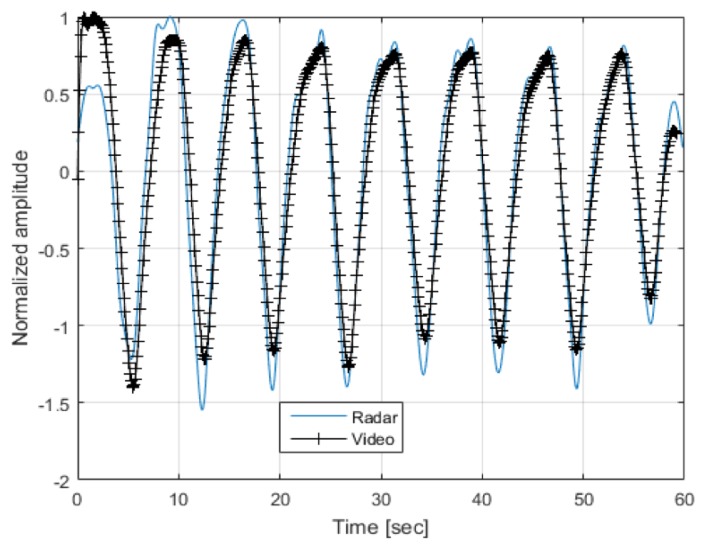
Radar and vision extracted breathing signals.

The range-Doppler map is searched for peaks using a constant false alarm rate (CFAR) detector, the largest peak inside the respiration frequency band is declared as the breathing frequency and is further validated by finding at least one more harmony (i.e., $2f_{b}$, $3f_{b}$ etc.) in the spectrum. This frequency value is then introduced as another input to the fusion algorithm. An example of the radar extracted and a vision extracted breathing signal is depicted in Figure 6. Note that the signals are highly correlated.

### 4.7. Fusing the Estimated Breathing Frequencies from the Rgbd Sensor and the Radar

The vision and radar based estimated frequencies that survived the tests described above, are collectively introduced into the maximum likelihood fusion algorithm. Each individual estimator ${\hat{\omega }}_{b_{i}}$, for each signal *i*, is associated with its own estimation error variance $\sigma_{\omega_{i}}^{2}$. These errors are assumed (An assumption that was validated in our experiments.) to be zero-mean Gaussian random variables. Thus, we can formulate the problem as follows,

(9) $$\begin{array}{ccc} {\hat{\omega }}_{b_{0}} & = & \omega_{b}+w_{0}\\ {\hat{\omega }}_{b_{1}} & = & \omega_{b}+w_{1}\\ \vdots \\ {\hat{\omega }}_{b_{N_{s}-1}} & = & \omega_{b}+w_{N_{s-1}}, \end{array}$$

where $w_{i}∼N\left(0,\sigma_{\omega_{i}}^{2}\right)$ and $\omega_{b}$ is the true parameter value.

Writing the same in a vector-matrix form

(10) $${\hat{\omega }}_{b}=1\omega_{b}+w,$$

where 1 is a $N_{s}\times 1$ vector of ones, and $w∼N\left(0,R\right)$ and $R=diag\left(\sigma_{\omega 0}^{2},\sigma_{\omega 1}^{2},\cdots ,\sigma_{\omega N_{s}-1}^{2}\right)$ is the error covariance matrix.

The solution is given by [19] (pp. 225–226)

(11) $$\begin{array}{ccc} {\hat{\omega }}_{b_{ML}} & = & {\left(1^{T}R^{-1}1\right)}^{-1}R^{-1}1^{T}{\hat{\omega }}_{b}\\ & = & \frac{1}{\sum_{i=0}^{N_{s}-1}\frac{1}{\sigma_{\omega i}^{2}}}\sum_{i=0}^{N_{s}-1}\frac{{\hat{\omega }}_{b_{i}}}{\sigma_{\omega i}^{2}}, \end{array}$$

where $N_{s}\leq 3N+1$ is the number of estimators that have survived and are participating in this last fusion step. This algorithm yields a refined estimate of the fundamental breathing frequency.

## 5. Experiments

The following sections describe the experiments and their results. The first batch of experiment was done on three adults and two babies; the same subjects as in [9] for comparison purposes. The second batch of experiments was done on 10 healthy adults 20 to 45 years of age. All subjects gave their written consent as well as no underlying respiratory issues were reported.

### 5.1. Comparing Obtained Results to Our Previous Work

The algorithm was tested on three adults and two babies (All of which gave their consent to participate). The true breathing rate was estimated manually from the 10 s long video feed. The results are given in Table 2. After 10 s of starting of an experiment, we get a maximal error of 0.5 BPM, which is twice as accurate as the framework and hardware we proposed in [9].

Futhermore, the true rate for each subject was compared against the high-complexity ML estimator that solves for all parameters in (2), with the addition of the radar extracted breathing rate using the whole 10 s. This approach is optimal so it is used as a lower bound of the estimation error. As seen from Table 2, the optimal ML estimator yields a maximal error of 0.15 BPM. Moreover, the largest deviation of the proposed algorithm from the optimal ML is only 0.38 BPM.

### 5.2. More Experiments

Nine more subjects were recruited to run more experiments. The subjects are healthy/non-smoking females and males with no respiratory underlying medical conditions. The experiments were divided to two phases. The first phase, involving all 9 subjects, was conducted in a set-up as depicted in Figure 2. Two minutes of breathing were recorded. Each recording was split into 12 non-overlapped sections of 10 s to cover the whole two minutes. The maximum error and the mean error (over these 12 sections) is reported in Table 3.

### 5.3. Extracted Signal Fidelity

**Figure 7:**
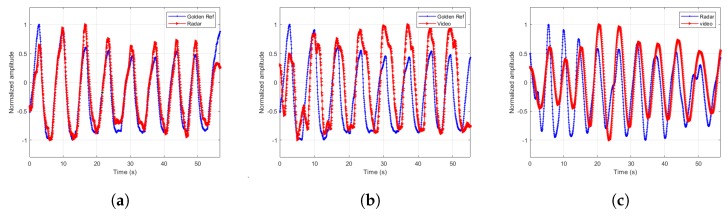
Extracted signals comparisons for breathing rate of 9.37 BPM. (**a**) Radar and ground truth signals. (**b**) Depth and ground truth signals. (**c**) Radar and depth signals.

**Figure 8:**
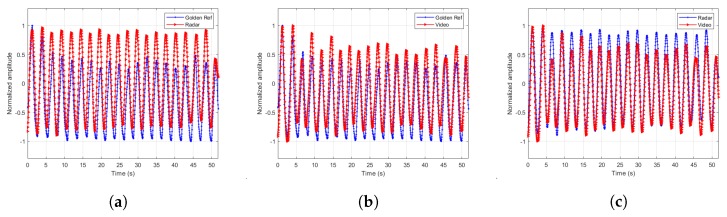
Extracted signals comparisons for breathing rate of 23.43 BPM. (**a**) Radar and ground truth signals. (**b**) RGB and ground truth signals. (**c**) Radar and RGB signals.

**Figure 9:**
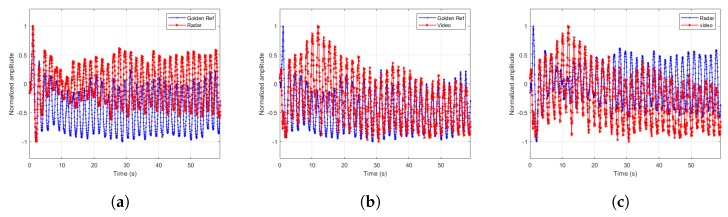
Extracted signals comparisons for breathing rate of 33.98 BPM. (**a**) Radar and ground truth signals. (**b**) RGB and ground truth signals. (**c**) Radar and RGB signals.

**Figure 10:**
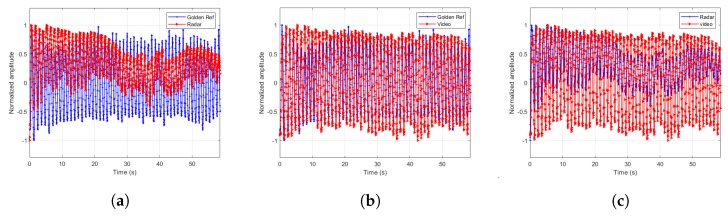
Extracted signals comparisons for breathing rate 56.25 BPM. (**a**) Radar and ground truth signals. (**b**) RGB and ground truth signals. (**c**) Radar and RGB signals.

The extracted signals are paramount to the estimator’s accuracy. Therefore, we designed a few more experiments to demonstrate the extracted signal fidelity. In these experiments, we chose one male subject of age 43 and asked him to breathe at different breathing rates according to the cadence of a metronome. We recorded the torso movement through time using Neulog’s Respiration Monitor Belt logger NUL-236 [20] to be used as a golden reference or ground truth. Except for visually confirming high correlation between the video, radar and ground truth, as depicted in Figure 7, Figure 8, Figure 9 and Figure 10, we also ran a sample by sample sliding window of 10 s, on approximately 60 s of recording. On each 10 s window, we performed DFT and compared the peak frequency value between the radar, RGB, and depth signals. We report the accuracy results in Table 4. As can be seen, the fidelity of the signals extracted is very high, with maximum error of 0.1731 BPM (error of 0.3%) for the scenario in which the subject was breathing extremely fast.

## 6. Conclusions

This paper presents a novel, illumination insensitive system and set of algorithms from which a human breathing rate is remotely extracted, using a radar and a RGBD camera. The vision-based algorithm is estimating the breathing rate by tracking in three dimensions (x, y, depth) points of interest in the frame coupled with the radar-based estimation, which are then fused into a single more accurate estimation using a ML approach. Experiments were done on 14 subjects and show over twofold improvement comparing to the results we reported at [9], which were RGB vision-based only. Moreover, the extracted signals (from the RGD, depth, and radar modalities) fidelity was qualitatively and quantitatively inspected and yielded very positive, high fidelity results.

## Figures and Tables

**Table 1:** Radar parameters.

	Value	Units	Comment
**Pulse Repetition Frequency**	40.5	MHz	
**Center frequency**	7.29	GHz	
**Bandwidth**	1.5	GHz	
**Peak pulse power**	–0.7	dBm	
**Frame rate**	∼10	Hz	∼70 dB processing gain

**Table 2:** Experiments results.

Subject Gender [m/f]	Subject Age [years]	Maximum Error- Proposed Algorithm [BPM]	Maximum Error- Optimal Algorithm [BPM]
m	40	0.26	0.1
m	50	0.3	0.12
m	30	0.4	0.15
m	2	0.4	0.2
f	0.5	0.5	0.12

**Table 3:** Experiments results.

Subject Gender [f/m]	Subject Age [years]	Mean Breathing Rate [BPM]	Mean Error [BPM]	Mean Error [% of Breathing Rate]
f	25	14	0.27	1.92
f	28	13	0.33	2.53
f	31	16	0.29	1.81
m	35	16	0.29	1.81
f	38	14	0.39	2.78
f	39	14	0.31	2.21
m	41	15	0.40	2.66
m	43	13	0.39	2.92
m	43	17	0.41	2.41

**Table 4:** Signal fidelity experimental results.

Breathing Rate (BPM)	Mean Radar Error (BPM)	Mean RGB Error (BPM)	Mean Depth Error (BPM)
9.37	0	0	0.0020
23.43	0	0	0.0019
33.98	0.0191	0.0191	0.0193
56.25	0.1254	0.1688	0.1731
